# R2KAN-U-Net: A Novel Architecture Integrating Kolmogorov–Arnold Networks with Residual U-Net for Robust Traffic Sign Segmentation

**DOI:** 10.3390/s26123797

**Published:** 2026-06-15

**Authors:** Taha Ben-Abbou, Houda El Omrani, Khalid El Fazazy, Mohamed Adnane Mahraz, Hamid Tairi, Jamal Riffi

**Affiliations:** L3IA—Laboratory of Computer Science, Innovation, and Artificial Intelligence, Faculty of Sciences Dhar El Mahraz, Sidi Mohamed Ben Abdellah University, Fez 30003, Morocco; houda.elomrani@usmba.ac.ma (H.E.O.); mohamed.mahraz@usmba.ac.ma (M.A.M.);

**Keywords:** traffic sign segmentation, autonomous driving, intelligent transportation systems, semantic segmentation, Residual–Recurrent U-Net, Kolmogorov–Arnold networks, multi-scale feature fusion, low-light road scenes

## Abstract

Traffic sign segmentation is a fundamental component of intelligent transportation systems and autonomous driving, where reliable pixel-level perception is required under challenging real-world conditions such as illumination variations, occlusion, scale diversity, and complex urban backgrounds. In this work, we propose Residual–Recurrent Kolmogorov–Arnold Network U-Net (R2KAN-U-Net), where “R2” denotes the integration of residual convolutional learning and recurrent KAN-based feature refinement. The proposed architecture combines residual U-Net feature extraction, multi-scale KAN fusion, and recurrent KAN refinement to improve pixel-level traffic sign segmentation under challenging road-scene conditions. The proposed framework integrates three complementary components: (1) residual convolutional blocks for stable feature propagation; (2) a multi-scale KAN fusion bottleneck for capturing contextual information at different receptive fields; and (3) recurrent KAN refinement modules for iterative enhancement of discriminative features. Unlike conventional convolutional architectures, the proposed KAN-based formulation replaces linear transformations with learnable univariate functions, enabling adaptive nonlinear feature modeling. We conduct extensive experiments on a custom dataset containing 9300 annotated urban traffic scene images, as well as on the ADE20K and Cityscapes benchmarks. On the custom dataset, the proposed R2KAN-U-Net achieved a Dice coefficient of 0.92 and an IoU score of 0.89, providing a strong accuracy–efficiency trade-off for traffic-sign foreground segmentation. It achieves competitive segmentation accuracy compared with recent CNN-, transformer-, and state-space-based segmentation models while using fewer parameters and lower computational cost. Additional low-light experiments demonstrate improved segmentation stability, with R2KAN-U-Net achieving the highest low-light Dice score of 0.88 and a competitive low-light IoU of 0.79. Furthermore, the proposed architecture maintains competitive computational efficiency with only 24 M parameters, 44.8 G FLOPs, and near-real-time inference at 13 ms per image. The experimental results demonstrate that integrating KAN-based function-space learning with residual and multi-scale feature refinement provides an effective and computationally efficient solution for robust traffic sign segmentation in complex driving environments.

## 1. Introduction

Semantic segmentation is a fundamental task in computer vision that aims to assign a semantic label to each pixel in an image. The extent of granularity allows a high level of scene interpretation, and segmentation is therefore essential in many applications such as medical imaging, autonomous driving, remote sensing, and others [1].

Among these applications, traffic sign segmentation has become increasingly important in intelligent transportation systems (ITS) and autonomous driving. At the pixel level, the ability to recognize traffic signs is the core part of providing trust and safety to driver-assistance technologies and completely automated driving. Traffic signs provide critical information for navigation, speed regulation, and hazard awareness in autonomous driving systems, assisting them to increase or reduce their speed, navigate through intersections and respond to potential hazards when seen and located in time [2].

Segmentation of road traffic signs has remained a complicated task since there are few intrinsic demerits. Traffic sign segmentation remains challenging due to variations in shape, size, color, illumination, and environmental conditions [3]. Deep neural networks and convolutional neural networks (CNNs) have transformed image segmentation because the end-to-end means learning can be done with the raw image and an output of the segmented image.

Amongst all of the architectures, U-Net and its derivatives have stood the test of time, as their encoder–decoder network design has proven to provide optimal performance, as deep semantic features can easily be merged with high-resolution spatial data by simply concatenating the skip connections. U-Net, first designed to be used in biomedical image analysis, is adopted for use in parsing urban scenes and traffic sign segmentation. Although it has some strengths, traditional U-Net structures have problems with long-range contextual dependency and multi-scale representation of traffic signs.

The proposed architecture integrates residual convolutional learning, recurrent KAN [4] refinement, and multi-scale feature fusion within a unified U-Net framework. Specifically, residual convolutional blocks are used in the encoder–decoder backbone, while recurrent KAN refinement modules and multi-scale KAN fusion are incorporated into the bottleneck to enable adaptive function-space feature transformation beyond conventional convolutional representations. This design aims to improve traffic sign segmentation accuracy under challenging real-world conditions while maintaining computational efficiency.

In this work, we investigate an enhanced U-Net architecture for robust traffic sign segmentation. We conduct extensive experiments on a custom traffic-sign dataset and public benchmark settings, provide qualitative and quantitative evaluations, and compare the proposed method with classical and contemporary segmentation baselines. The overall goal is to support safer and more reliable traffic-scene understanding for intelligent transportation and autonomous driving applications [5].

### Novelty Statement

Following are the major contributions of this work:

1. We introduce a function-space segmentation framework by integrating Kolmogorov–Arnold Networks (KANs) into a U-Net architecture, enabling adaptive nonlinear feature transformation beyond conventional activation-based representations.
2. We propose a multi-scale KAN bottleneck module that captures feature interactions across different receptive fields through parallel functional transformations.
3. We design a recurrent residual KAN refinement mechanism that iteratively enhances feature representations, improving segmentation accuracy for small and occluded traffic signs.
4. We conduct extensive experiments on a custom dataset and public benchmarks, including ADE20K and Cityscapes, demonstrating that the proposed architecture achieves competitive performance against representative classical and contemporary segmentation baselines.
5. We provide ablation studies validating the individual and joint contributions of the proposed modules.

## 2. Related Work

Traffic sign perception includes several related but distinct tasks. Traffic sign detection aims to localize signs in an image, usually through bounding boxes or candidate regions. Traffic sign classification/recognition assigns a semantic class label, such as speed limit or stop sign, to a detected or cropped sign. In contrast, traffic sign segmentation, which is the focus of this work, predicts a pixel-level foreground mask that delineates the traffic-sign region from the background. Therefore, recognition and classification methods are reviewed as related traffic-sign perception approaches, but they are not used as direct quantitative baselines because they optimize image-level or object-level accuracy rather than pixel-level metrics such as Dice coefficient and IoU.

Earlier approaches to traffic sign detection and segmentation were highly reliant on hand-designed features like color and shape descriptors and HSV space color thresholds but were affected easily by occlusion, changes in lighting conditions and the presence of complicated surroundings [6]. The emergence of deep learning and convolutional neural networks significantly transformed the field. The U-Net architecture, proposed by Ronneberger et al. [7], has created a powerful encoder–decoder framework, whose skip connections maintain spatial information between layers in a given image. The success of its application in biomedical image segmentation soon made itself felt in the road-scene parsing scenario.

UNet++ [8] has extended this architecture to the nested and dense skip pathways to propagate features better and regionally represent them at different scales. However, in deeper networks, there is the problem of vanishing gradients, which was solved by ResNet [9] with identity-based residual links. Along with allowing deeper architectures, residual learning has heralded much faster convergence, in addition to better model generalization.

In recent years, transformer-based segmentation models have achieved state-of-the-art performance by effectively modeling long-range dependencies and global context. SegFormer [10] employs a hierarchical transformer encoder that enables a balance between local and global features, and is computationally efficient. Mask2Former [11] incorporates semantic, instance and panoptic segmentation into one model, and shows competitive performance across several benchmarks. Other new transformer-based models, e.g., Swin-UNet [12] and Segmenter [13], further improve the performance of segmentation by means of adopting multi-head self-attention to extract global features, which has achieved significant performance gain over conventional, CNN-based methods and real strengths in urban street scene segmentation. It can be seen in these models that it is vital to capture the global context of such environments without losing any of the local spatial detail and this is especially true of traffic sign segmentation in a cluttered setting.

In addition to residual learning, it is necessary to incorporate spatial context. PSPNet [14] applied pyramid pooling to get global scene information whilst DeepLabV3+ [15] used atrous spatial pyramid pooling (ASPP) in conjunction with depthwise separable convolutions to maximize feature extraction at scale. This trend was further continued on by HRNet [16] which kept the resolutions high by the use of parallel branches, which proved to be very useful in the segmentation of small objects, for example, distant or slightly occluded traffic signs.

Attention mechanisms were also a response to the necessity of focusing the computation on what proves most interesting in an input. In Attention U-Net [17], the author introduced attention gates, and such gates selectively modulated elements of skip connections and enabled the model to filter out unnecessary background noise. Subsequent architectures introduced recurrent mechanisms to iteratively refine segmentation features, such as R2AU-Net [18], so that the segmentations in the previous frame can be iterated and refined and in order to better represent the surroundings so that this can be carried out more effectively.

Among some of the recent inventions is the adaptivity of the activation functions. Nonlinearities, in turn, were replaced with kernel functions in the Kernel Adaptive Networks introduced by Bietti and Mairal [19], in which the network learns the activation behavior that suits the structure of the data. This strategy was later generalized in [20], which adapted kernel learning to a more general nonlinear family of functions and also showed better stability and performance in dense prediction tasks.

Recent studies have explored Kolmogorov–Arnold Networks in segmentation and perception tasks beyond conventional MLP-style function approximation. KMS-Net [21] applies KAN-based multi-scale attention for cardiac segmentation, combining spline-based KAN layers, multi-scale attention, and long-range modeling components for medical image analysis. KR-Mamba [22] integrates KAN with reverse-channel attention and Mamba-style state-space modeling for medical image segmentation. SA-U-KAN [23] combines convolutional feature extraction, spatial attention, KAN-based tokenization, and U-Net-style decoding for optic disk and cup segmentation. KAN has also been explored outside 2D image segmentation, such as in LiDAR odometry denoising under high-reflectivity noise [24]. These studies demonstrate the growing relevance of KAN-based function modeling across medical imaging and perception tasks.

Compared with these works, R2KAN-U-Net is designed specifically for traffic-sign foreground segmentation in road scenes. Instead of focusing on cardiac structures, optic disk/cup boundaries, or LiDAR point-cloud denoising, the proposed architecture targets small, cluttered, and illumination-sensitive traffic-sign regions. Its main design difference is the combination of residual U-Net feature extraction, multi-scale KAN fusion, and recurrent KAN refinement for pixel-level traffic-sign mask prediction.

Several surveys have summarized segmentation architectures. Minaee et al. [25] provide a comprehensive taxonomy of semantic segmentation methods across application areas, whereas Litjens et al. [26] review deep learning methods in medical image analysis, many of which are relevant to road-scene understanding. Multi-column deep neural networks have also been applied to traffic-specific tasks, where Ciresan et al. [27] also applied the collective learning approach to traffic sign classification. Zhang and Shao proposed an enhanced U-Net to segment traffic signs applied with special loss functions and additional attention modules. There have also been attempts at alleviating the need for annotation by domain adaptation [28] and self-supervised learning [29] in order to generalize to other geographic areas or lighting conditions.

Segmentation is an important task in the field of image processing where transformer-based segmentation models have achieved great success in the past several years. SegFormer proposed a powerful transformer architecture with both local and global contexts, which reduces the computational overhead typically associated with pure transformer architectures, and it achieved state-of-the-art results on the ADE20K [30] and Cityscapes [31] benchmarks. This transformer-based model uses a simple MLP decoder that makes it computationally efficient as compared to other complex transformer-based models.

Similarly, Goel and Koundal (2025) combined instance segmentation and MaskFormer [32] EfficientNet in counting crowds. The MaskFormer method takes advantage of the masked attention system which can dramatically improve the precision of segmentation in difficult images like crowded or occluded situations. This is needed in work requiring a high level of detail such as in counting crowds where segmentation of individuals is necessary to accurately analyze and make independent decisions.

Strudel et al. proposed Segmenter, a Vision Transformer-based model for semantic segmentation that captures global context and predicts dense pixel-level labels using transformer-based decoding. The method achieved strong results on ADE20K, Pascal Context, and Cityscapes, demonstrating the effectiveness of pure transformer architectures for segmentation tasks.

Recent research also highlights the hybrid systems that combine CNN and transformer-based models to deliver efficient segmentation. TransUNet [33], for example, employs a hybrid of transformers and U-Net, which provide both local and global information when making predictions.

There has also been some interest in the use of multi-scale and multi-task learning models, which can be seen to support simultaneous business across a number of segmentation tasks. Examples include Panoptic-DeepLab [34], which integrates instance segmentation with semantic segmentation to offer a unified solution to pixel-wise object classification and detection, which is also applicable in autonomous driving, i.e., road sign detection.

In spite of the encouraging performance of these models, there are a number that are computationally demanding and hence unsuitable in real-time applications. However, the proposed R2KAN-U-Net incorporates residual convolutional blocks, multi-scale fusion, and recurrent KAN refinement modules to provide efficient solutions to traffic sign segmentation, especially small and occluded ones. In comparison to SegFormer and Mask2Former, which use transformers to obtain a global context, R2KAN-U-Net achieves good performance at low cost since it balances local feature refinement and the adaptive iterative learning process.

## 3. Proposed Approach

### 3.1. Architecture Overview

This research introduces the Residual–Recurrent Kolmogorov–Arnold Network U-Net (R2KAN-U-Net), a novel deep learning architecture that synergistically combines the spatial feature extraction capabilities of U-Net with the function approximation prowess of Kolmogorov–Arnold Networks (KANs). The proposed model addresses the inherent limitations of conventional convolutional neural networks in capturing complex nonlinear relationships within traffic sign segmentation tasks by incorporating learnable univariate functions through KAN layers, which replace conventional linear transformations and enable adaptive nonlinear feature modeling.

### 3.2. Core Architectural Components

The architecture consists of four main components: (1) an encoder pathway with residual convolutional blocks; (2) a specialized bottleneck processing module incorporating multi-scale KAN transformations; (3) a decoder pathway with skip connections; and (4) recurrent KAN blocks for iterative feature refinement.

#### 3.2.1. Residual Convolutional Blocks

The foundation of our encoder–decoder structure employs residual convolutional blocks that mitigate the vanishing gradient problem commonly encountered in deep segmentation networks. Each residual block consists of two consecutive 3 × 3 convolutional layers with ReLU activations, followed by batch normalization. The residual connection is implemented through element-wise addition, with dimensional matching achieved via 1 × 1 convolutions when necessary:

$$x_{out}=F\left(x,\left\{W_{i}\right\}\right)+x$$

where $F\left(x,\left\{W_{i}\right\}\right)$ represents the convolutional transformation and x denotes the identity mapping. This formulation enables the network to learn residual mappings, facilitating gradient flow and improving convergence characteristics.

#### 3.2.2. Kolmogorov–Arnold Network Integration

The proposed architecture incorporates Kolmogorov–Arnold Networks (KANs) within the bottleneck to enhance nonlinear feature modeling. KANs are inspired by the Kolmogorov–Arnold representation theorem, which states that any multivariate continuous function can be decomposed into a finite sum of univariate functions.

Unlike conventional neural networks, which model feature transformations through linear weights followed by fixed activation functions, KAN replaces these linear transformations with learnable univariate functions defined on edges. This formulation enables the network to directly model complex feature interactions in function space, providing greater flexibility and interpretability in representing nonlinear relationships.

Formally, a KAN layer can be expressed as

$$f(x)=\sum_{i}\varphi_{i}\left(\sum_{j}\psi_{ij}\left(x_{j}\right)\right)$$

where ψᵢⱼ and φᵢ are learnable univariate functions, implemented using B-spline basis functions.

In practice, these functions are implemented using B-spline basis functions, allowing smooth and adaptive approximation of nonlinear mappings. In this work, we employ cubic B-splines with a fixed number of control points (e.g., 16 per function) and uniform grid initialization, enabling the model to learn task-specific nonlinear transformations tailored to traffic sign features.

This design allows the proposed architecture to capture structured and fine-grained visual patterns more effectively than standard convolutional representations, particularly in challenging scenarios involving small objects, occlusion, and complex backgrounds.

#### 3.2.3. Multi-Scale Feature Processing

To effectively capture features across different spatial extents, we introduce a multi-scale KAN-based fusion module within the bottleneck layer. This module is designed to approximate multiple receptive fields, enabling the model to simultaneously encode fine-grained local details and broader contextual information.

**Figure 1:**
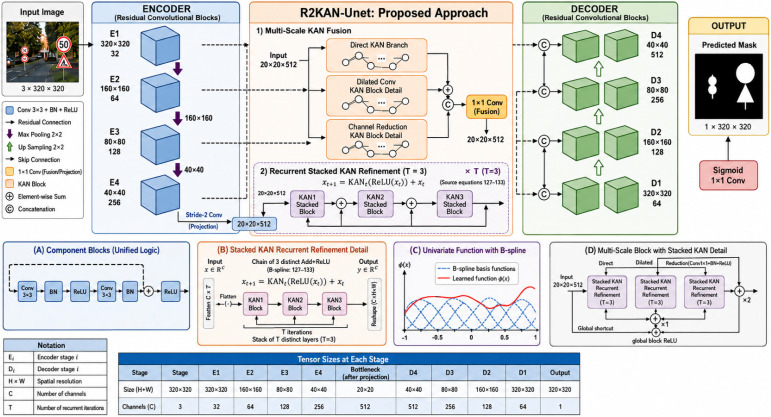
Proposed approach of R2KAN-U-Net.

Conceptually, the proposed multi-scale design corresponds to three receptive fields (3 × 3, 5 × 5, and 7 × 7), as illustrated in Figure 1. Instead of explicitly using large convolutional kernels, which would significantly increase computational cost, these receptive fields are approximated through efficient preprocessing operations applied prior to the KAN transformation.

Specifically, the input feature map X is processed through three parallel branches:

1. Local Branch: The input features are directly passed through a KAN transformation, preserving fine spatial details and local structures.
2. Contextual Branch: A 3 × 3 convolution with dilation rate 2 is applied to expand the effective receptive field, approximating a 5 × 5 neighborhood before KAN processing.
3. Global Branch: A 1 × 1 convolution is employed for channel projection and feature compression, followed by KAN transformation to capture more global and abstract feature interactions.

The outputs of these branches are fused via element-wise summation to produce a unified multi-scale feature representation. Formally, the fused feature map is defined as

$$X_{multi}=\sum_{i=1}^{3}{KAN}_{i}(F_{i}\left(X\right))$$

where

- $F_{1}(X)=X$;
- $F_{2}(X)=Conv_{dilated}(X)$;
- $F_{3}(X)=Conv_{proj}(X)$.

Conv_dilated(·) denotes a 3 × 3 convolution with dilation rate r = 2, and Conv_proj(·) denotes a 1 × 1 convolution used for channel-wise projection.

#### 3.2.4. Residual KAN Blocks

Individual KAN processing units incorporate residual connections to maintain gradient flow and prevent feature degradation:

$$X_{kan}=KAN\left(x_{flat}\right)+x_{flat}$$

where $x_{flat}$ represents the spatially flattened input tensor. This residual formulation ensures that the KAN layers can learn incremental improvements to the feature representation without losing essential spatial information.

#### 3.2.5. Recurrent Residual KAN Processing

For enhanced feature learning capacity, the architecture implements stacked KAN layers with recurrent residual connections. The stacked KAN block processes features through multiple sequential KAN transformations, each incorporating its own residual connection:

$$x_{0}=Fllaten(x_{spatial})\;x_{1}=KAN_{1}\left(x_{0}\right)+x_{0}\;x_{2}=KAN_{2}\left(RELU\left(x_{1}\right)\right)\;+x_{1}\;\ldots \;X_{n}=KAN_{n}\left(RELU\left(X_{n-1}\right)\right)+X_{n-1}$$

where n represents the number of stacked layers (typically 3), and each KANᵢ denotes an independent KAN transformation. This recurrent residual formulation allows the network to iteratively refine feature representations while maintaining gradient flow through multiple processing stages. The final output is then reshaped back to spatial dimensions:

$$X_{output}=Reshape(X_{n},\;\left(H,W,C\right))$$

This stacked design enables deeper feature abstraction while preserving stable gradient propagation through residual learning, particularly beneficial for capturing complex traffic sign patterns and subtle discriminative features.

### 3.3. Network Architecture Design

#### 3.3.1. Encoder Pathway

The encoder follows a traditional U-Net structure with four downsampling stages, each reducing spatial dimensions by a factor of two while doubling the channel count. Each encoder level employs residual convolutional blocks to extract hierarchical features:

- Level 1: 32 filters, spatial resolution 320 × 320;
- Level 2: 64 filters, spatial resolution 160 × 160;
- Level 3: 128 filters, spatial resolution 80 × 80;
- Level 4: 256 filters, spatial resolution 40 × 40.

Max pooling operations facilitate downsampling while preserving important spatial features.

#### 3.3.2. Bottleneck Processing

The bottleneck layer operates at 20 × 20 spatial resolution with 512 channels, incorporating both residual convolutional processing and multi-scale KAN transformation. The spatial dimensions are dynamically calculated based on input size to accommodate various image resolutions:

$$bottleneck_{spatial}=(H/16,W/16)$$

where H and W represent input height and width respectively.

#### 3.3.3. Decoder Pathway

The decoder employs transpose convolutions for upsampling, with skip connections from corresponding encoder levels to preserve spatial details. Each decoder level combines upsampled features with encoder features through concatenation, followed by residual convolutional processing to refine the merged representations.

### 3.4. Loss Function and Training Strategy

To optimize segmentation performance, we employ a composite loss function combining binary cross-entropy (BCE) and Dice loss:

$$L\;=\;\lambda_{1}L_{BCE}\;+\;\lambda_{2}L_{Dice}$$

where

$$L_{BCE}=-\frac{1}{N}\sum \left[y\log\left(\hat{y}\right)+\left(1-y\right)\log\left(1-\hat{y}\right)\right]$$

$$L_{Dice}=1-\frac{2\sum_{i}y_{i}{\hat{y}}_{i}+\varepsilon }{\sum_{i}y_{i}+\sum_{i}{\hat{y}}_{i}+\varepsilon }$$

Here, yᵢ ∈ {0, 1} denotes the ground-truth label of pixel i, ${\hat{y}}_{i}$ ∈ [0, 1] denotes the predicted foreground probability, N is the total number of pixels, and ε is a small constant used to ensure numerical stability. In our experiments, λ_1_ = 0.5 and λ_2_ = 0.5.

The BCE term encourages pixel-wise classification accuracy, while the Dice term improves foreground–background overlap, which is particularly important for traffic sign segmentation where foreground pixels are often much fewer than background pixels.

### 3.5. Architectural Advantages

The proposed R2KAN-U-Net architecture offers several key advantages:

1. Enhanced Nonlinear Modeling: The integration of Kolmogorov–Arnold Networks (KANs) enables adaptive nonlinear feature transformation by replacing conventional linear weights with learnable univariate functions. This formulation allows the model to explicitly capture complex feature interactions in function space, improving its ability to represent fine-grained traffic sign patterns.
2. Multi-Scale Feature Extraction: The multi-scale KAN fusion module captures feature representations at different receptive fields, enhancing robustness to variations in traffic sign size, scale, and surrounding context.
3. Gradient Flow Optimization: Residual connections throughout the network facilitate stable gradient propagation, enabling efficient training of deeper architectures without performance degradation.
4. Spatial Coherence Preservation: The combination of convolutional spatial processing and KAN-based functional modeling preserves spatial structure while enhancing discriminative feature representation.

Overall, this architectural design effectively addresses key challenges in traffic sign segmentation, including varying illumination conditions, diverse sign geometries, and complex background environments, while maintaining computational efficiency suitable for real-world deployment.

The complete architecture is illustrated in Figure 1.

## 4. Experimental Results

### 4.1. Dataset Description

We constructed a dataset consisting of 9300 high-resolution urban road images with pixel-level annotations for traffic sign segmentation. The dataset includes diverse environmental conditions such as varying illumination, weather, occlusion, and background complexity.

Annotation Protocol:

All images were manually annotated using a polygon-based labeling tool. Each image was independently reviewed by two annotators, and discrepancies were resolved through consensus to ensure annotation quality.

Class Distribution:

The dataset includes three main categories of traffic signs:

- Warning signs: 35%;
- Regulatory signs: 40%;
- Prohibitory signs: 25%.

Dataset Split:

The dataset is divided into:

- 70% training (6510 images);
- 15% validation (1395 images);
- 15% testing (1395 images).

To ensure generalization, scenes are non-overlapping across splits.

**Figure 2:**
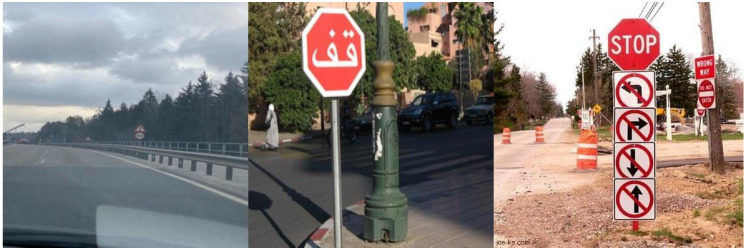
Random samples from custom dataset.

We plan to release a representative subset of the dataset along with annotation guidelines to support reproducibility. Here, are some examples of our dataset in Figure 2:

### 4.2. Experimental Setup

We conduct our experiments on a self-generated dataset consisting of 9300 annotated images of road traffic signs. The dataset encompasses a wide variety of traffic signs captured under diverse environmental conditions, including variations in lighting, occlusions, scale, and cluttered backgrounds. It covers different categories of traffic signs—warning, regulatory, and prohibitory—which are crucial for evaluating the segmentation accuracy of our model.

#### 4.2.1. Data Preprocessing and Augmentation

To ensure consistency and improve model robustness, we apply several preprocessing and augmentation strategies:

1. Image Scaling

We uniformly resize all images to 320 × 320 pixels. This resolution is selected as it provides a practical trade-off between computational efficiency and segmentation accuracy.

2. Normalization

We normalize pixel intensity values to the range **0–1**. This step facilitates faster convergence and stabilizes the training process.

3. Data Augmentation

To enhance generalization and reduce the risk of overfitting, we employ multiple augmentation techniques, including:

▪ Random horizontal flipping;
▪ Random rotations up to ±15°;
▪ Adjustments of contrast and brightness;
▪ Padding combined with random cropping.

The dataset is split into 70% training, 15% validation, and 15% testing, corresponding to 6510, 1395, and 1395 images, respectively. This split enables reliable evaluation on unseen samples while ensuring sufficient training diversity. We train the model using the Adam optimizer with a learning rate of 1 × 10^−4^ and optimize a combined binary cross-entropy and Dice loss to address class imbalance. We use a batch size of 16 and train for up to 400 epochs on an NVIDIA A100 GPU. Early stopping based on validation accuracy is used to avoid overfitting, and the best model is saved according to the validation score. The training configuration is listed in Table 1.

#### 4.2.2. Evaluation Metrics

➢ Dice Coefficient: Measures overlap quality between predicted P and ground-truth G masks:

$$Dice\left(P,G\right)=\frac{2|P\cap G|}{|P|+|G|}$$

where

- ∣P ∩ G∣ number of pixels correctly predicted as foreground (intersection).
- ∣P∣: number of foreground pixels in prediction.
- ∣G∣: number of foreground pixels in ground truth.
- Range: [0, 1], higher is better.

➢ Intersection-over-Union (IoU): Evaluates boundary delineation robustness:

$$IoU\left(P,G\right)=\frac{|P\cap G|}{|P\cup G|}=\frac{|P\cap G|}{|P|+|G|-|P\cap G|}$$

where

- ∣P ∪ G∣: union of predicted and ground-truth pixels.
- Range: [0, 1], higher indicates better overlap.

➢ Pixel Accuracy: Assesses overall classification correctness:

$$PA\left(P,G\right)=\frac{TP+TN}{TP+TN+FP+FN}$$

### 4.3. Experimental Results

To provide a more comprehensive and up-to-date assessment of the proposed architecture, we extend the comparative evaluation beyond classical CNN-based and early transformer-based segmentation models. In addition to U-Net, R2U-Net, DeepLabV3+, PSPNet, SegFormer, and Mask2Former, the revised comparison includes recent representative architectures: ConvNeXt V2 [35] + UPerNet [36], OneFormer [37], and SegMAN-S [38]. These additional baselines were selected to cover modern convolutional designs, universal transformer-based segmentation, and recent state-space/Mamba-inspired segmentation paradigms. All models are trained and tested on the same custom traffic-sign dataset under identical experimental settings, including the same train/validation/test split, input resolution, preprocessing strategy, augmentation pipeline, loss function, and evaluation metrics. Performance is evaluated using Dice coefficient, Intersection-over-Union (IoU), and pixel accuracy. These metrics jointly assess foreground–background overlap, segmentation boundary quality, and pixel-level classification correctness.

As shown in Table 2, classical segmentation models such as U-Net and R2U-Net provide useful historical reference points, achieving Dice scores of 0.82 ± 0.013 and 0.84 ± 0.011, respectively. DeepLabV3+ and PSPNet improve the IoU performance compared with the original U-Net, while transformer-based models such as SegFormer and Mask2Former achieve stronger segmentation accuracy, with IoU scores of 0.86 ± 0.010 and 0.87 ± 0.009, respectively. The newly added contemporary baselines further strengthen the comparative analysis. ConvNeXt V2 + UPerNet achieves a Dice coefficient of 0.88 ± 0.009 and an IoU score of 0.85 ± 0.011, demonstrating the effectiveness of modern convolutional feature extraction. OneFormer provides the strongest performance among the added contemporary baselines, with a Dice coefficient of 0.91 ± 0.006 and an IoU score of 0.88 ± 0.008, while SegMAN-S achieves competitive results with a Dice coefficient of 0.90 ± 0.007 and an IoU score of 0.87 ± 0.009.

Compared with both historical and contemporary baselines, the proposed R2KAN-U-Net achieves the best overall performance, with a Dice coefficient of 0.92 ± 0.005, an IoU score of 0.89 ± 0.007, and a pixel accuracy of 98.4 ± 0.2%. These results indicate that the integration of residual convolutional learning, multi-scale KAN-based feature fusion, and recurrent KAN refinement improves traffic sign segmentation accuracy. The improvement is particularly reflected in the Dice and IoU scores, suggesting that the proposed model provides more accurate foreground localization and better boundary delineation for traffic signs under challenging real-world conditions. Importantly, the inclusion of ConvNeXt V2 + UPerNet, OneFormer, and SegMAN-S provides a more current comparative context and demonstrates that R2KAN-U-Net remains competitive not only against classical CNN and transformer models, but also against more recent segmentation architectures.

Table 3 evaluates the robustness of R2KAN-U-Net under low-light conditions using both classical and contemporary segmentation baselines. All models exhibit reduced performance on the low-light subset, confirming the difficulty of segmentation under reduced illumination. Classical CNN-based models such as U-Net and DeepLabV3+ show larger performance drops, while SegFormer, Mask2Former, ConvNeXt V2 + UPerNet, OneFormer, and SegMAN-S provide stronger robustness. Among the evaluated models, R2KAN-U-Net achieves the best overall performance, with an overall IoU of 0.89 and Dice score of 0.92. Under low-light conditions, it obtains a Dice score of 0.88, which is the highest among all compared methods, and an IoU score of 0.79, which is competitive with the best-performing SegFormer result of 0.80. Compared with Mask2Former, OneFormer, and SegMAN-S, the proposed model achieves higher low-light Dice and IoU scores. These results indicate that the recurrent KAN refinement and multi-scale feature fusion improve foreground stability and help preserve traffic-sign boundaries under reduced illumination. Thus, R2KAN-U-Net demonstrates a strong robustness–accuracy trade-off compared with both historical and recent segmentation models.

Table 4 presents the computational efficiency comparison of the evaluated segmentation models in terms of parameter count, FLOPs, and inference time. The results show that R2KAN-U-Net achieves the lowest model complexity among all compared methods, with only 24 M parameters and 44.8 G FLOPs. This is lower than classical CNN-based models such as U-Net and DeepLabV3+, as well as more recent architectures including SegFormer, Mask2Former, ConvNeXt V2 + UPerNet, OneFormer, and SegMAN-S. Although U-Net has a slightly lower inference time of 12.5 ms compared with 13.0 ms for R2KAN-U-Net, the proposed model provides substantially higher segmentation accuracy, as reported in Table 2, while maintaining near-real-time performance.

Compared with contemporary baselines, R2KAN-U-Net is considerably more compact and computationally efficient. ConvNeXt V2 + UPerNet requires 60 M parameters and 101.3 G FLOPs, OneFormer requires 109 M parameters and 269.1 G FLOPs, and SegMAN-S requires 28 M parameters and 49.2 G FLOPs. In contrast, R2KAN-U-Net achieves higher segmentation accuracy with fewer parameters and lower FLOPs, demonstrating a favorable balance between accuracy, compactness, and computational efficiency. These results support the suitability of the proposed architecture for traffic sign segmentation scenarios where both segmentation quality and computational cost are important.

To further evaluate the generalization ability of the proposed R2KAN-U-Net beyond the custom traffic-sign dataset, we extended the evaluation to the ADE20K and Cityscapes benchmarks. Since the objective of this work is traffic-sign foreground segmentation, the ADE20K and Cityscapes experiments were conducted using the same binary foreground segmentation protocol adopted throughout the manuscript. Consequently, the reported Dice coefficient, foreground IoU, and pixel accuracy values should not be interpreted as standard full-scene multi-class IoU over all ADE20K and Cityscapes categories. Instead, these results are intended to measure cross-dataset generalization under a consistent task-specific foreground segmentation setting. For this benchmark experiment, we applied the same preprocessing strategy, input resolution, loss function, and evaluation metrics used in the custom dataset experiments. R2KAN-U-Net was compared with representative segmentation architectures, including U-Net, R2U-Net, DeepLabV3+, PSPNet, and Mask2Former. The main contemporary comparison with recent architectures is reported on the custom traffic-sign dataset in Table 2, while the ADE20K experiment in Table 5 provides additional evidence of the proposed model’s generalization capability under the adopted foreground segmentation protocol.

The results presented in Table 5 indicate that classical CNN-based models, such as U-Net and R2U-Net, perform reliably but fall short compared to more advanced transformer-based architectures. DeepLabV3+ and PSPNet achieve moderate improvements, while Mask2Former delivers stronger results with Dice scores of 0.826. Under the adopted foreground segmentation protocol, R2KAN-U-Net achieves the highest scores among the evaluated baselines on the ADE20K dataset, achieving a Dice score of 0.842, IoU of 0.744, and pixel accuracy of 96.1%. These improvements highlight the robustness of our network, which enhances feature representation and segmentation precision even on large-scale and diverse datasets.

To evaluate the effectiveness of our proposed R2KAN-U-Net architecture, we conducted comprehensive experiments on the Cityscapes dataset and compared its performance against established segmentation models. The comparison encompasses three key metrics: Dice coefficient for measuring overlap accuracy, IoU score for intersection-over-union evaluation, and pixel accuracy for overall classification precision.

The experimental results in Table 6 demonstrate that under the adopted binary foreground segmentation setting, R2KAN-U-Net achieves the highest Dice, IoU, and pixel accuracy among the evaluated baselines. Notably, our model attains the highest Dice coefficient of 0.921 and IoU score of 0.821, representing significant improvements over existing approaches. The pixel accuracy of 98.5 further validates the model’s robust segmentation capabilities on complex urban scene datasets.

### 4.4. Ablation Study

In order to quantify the effect of the major components of the R2KAN-U-Net architecture, we conducted ablation studies. Specifically, we analyzed the impact of removing the recurrent KAN refinement blocks, multi-scale fusion, and residual connections on the model’s performance. The results of the study are presented in Table 7.

**Recurrent Blocks**: The removal of recurrent KAN refinement results in a reduction in the Dice coefficient from 0.924 to 0.884 and a decline in IoU from 0.89 to 0.769. This indicates that recurrent refinement plays a crucial role in capturing fine-grained details and contextual relationships in traffic scenes. Traffic signs often appear under varying lighting conditions, partial occlusions, and scale variations; the recurrent mechanism enables iterative feature refinement, improving discrimination between visually similar signs and background elements. The observed performance degradation highlights the importance of iterative KAN-based refinement for robust segmentation in real-world driving environments.

**Multi-Scale Fusion**: Eliminating the multi-scale fusion module leads to a decrease in Dice (from 0.924 to 0.892) and IoU (from 0.89 to 0.777), with only a marginal drop in pixel accuracy. This demonstrates the importance of capturing features at multiple spatial resolutions. In practical scenarios, traffic signs may appear at different distances and scales, requiring the model to combine fine boundary information with broader contextual cues. The performance drop confirms that multi-scale feature fusion is essential for consistent segmentation across diverse conditions.

**Residual Connections:** The removal of residual connections produces the most significant individual performance degradation, with Dice decreasing to 0.876 and IoU to 0.753. Residual learning is critical for maintaining stable gradient propagation and preserving discriminative features across layers. In traffic sign segmentation, where subtle geometric and color patterns are important, residual connections ensure effective feature reuse and generalization. This result highlights their fundamental role in both training stability and segmentation accuracy.

**Combined Ablations:** To further investigate the interaction between architectural components, we evaluate configurations where multiple modules are removed simultaneously. When both recurrent refinement and multi-scale fusion are removed, performance drops more significantly (Dice = 0.861, IoU = 0.741), indicating that these two components provide complementary benefits. The absence of both mechanisms reduces the model’s ability to capture multi-scale context and refine features iteratively. The most severe degradation is observed when both residual connections and recurrent refinement are removed (Dice = 0.852, IoU = 0.732), demonstrating that stable feature propagation and iterative refinement jointly contribute to effective feature learning. These results confirm that the proposed modules are not independent; rather, they interact synergistically to enhance segmentation performance.

**Overall Implications:** Collectively, the ablation results confirm that each architectural component—recurrent KAN refinement, multi-scale fusion, and residual connections—plays a non-trivial role in improving segmentation performance. More importantly, the combined ablation analysis demonstrates that these components interact in a complementary manner, enabling the proposed R2KAN-U-Net to effectively handle key challenges such as scale variation, occlusion, and complex backgrounds. This synergy is essential for achieving robust and reliable performance in real-world traffic sign segmentation tasks.

To further verify the utility of the KAN component, we conducted a replacement ablation study in which the KAN bottleneck/refinement module was replaced with several non-KAN alternatives while keeping the same encoder–decoder backbone, input resolution, dataset split, training strategy, and loss function. The compared alternatives include a standard MLP bottleneck, SE attention, CBAM attention, a ConvNeXt-style convolutional refinement block, and a lightweight state-space/Mamba-style refinement block. This experiment is designed to determine whether the proposed KAN-based function-space modeling provides additional benefit beyond conventional nonlinear projection, attention-based recalibration, modern convolutional refinement, and state-space feature modeling.

As shown in Table 8, replacing the bottleneck/refinement module with MLP, attention-based, ConvNeXt-style, or state-space refinement modules improves the residual U-Net baseline to different degrees. The standalone KAN bottleneck provides only limited improvement in overlap-based metrics and does not by itself achieve the strongest performance. However, when KAN is integrated with multi-scale feature fusion and recurrent refinement, the full R2KAN-U-Net achieves the best overall results, with Dice = 0.924, IoU = 0.890, and pixel accuracy = 98.4%. These results suggest that the benefit of the proposed design comes from the combined architecture—multi-scale KAN fusion, recurrent KAN refinement, and residual learning—rather than from simply inserting a KAN bottleneck.

**Figure 3:**
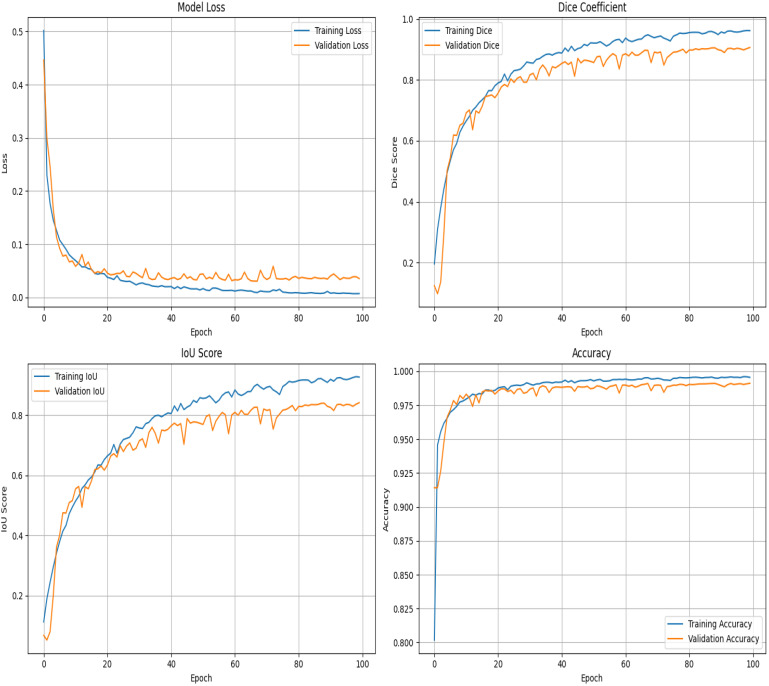
Training performance.

Figure 3 shows the training and validation performance of the proposed R2KAN-U-Net.

### 4.5. Real Results on Different Images

**Figure 4:**
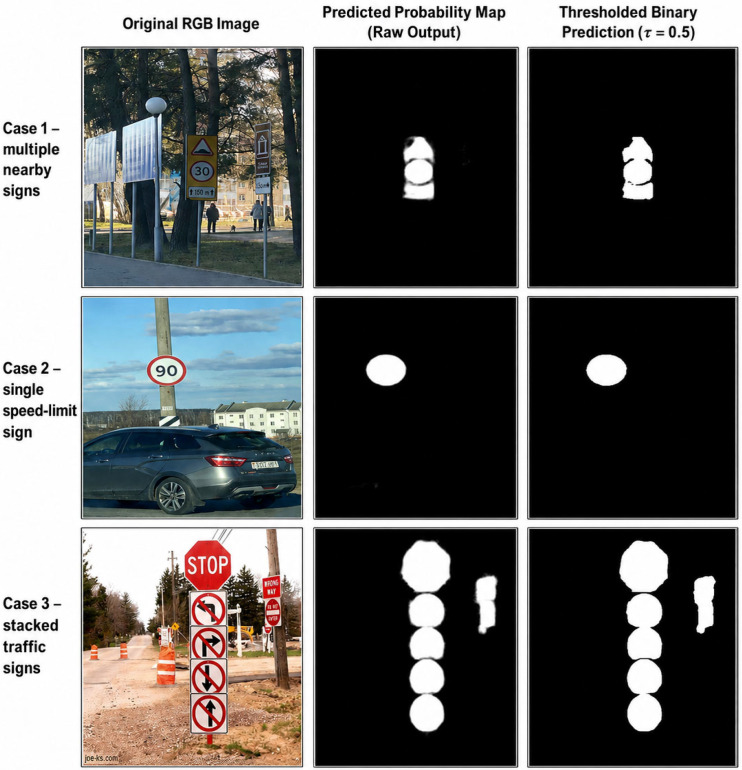
Segmentation test on real images.

Figure 4 presents qualitative segmentation examples obtained using the proposed R2KAN-U-Net on real traffic-scene images. The purpose of this figure is to clarify the complete visual segmentation process, from the original RGB input to the final binary segmentation output. The first column shows the original traffic-scene images, including multiple nearby signs, a single speed-limit sign, and stacked traffic signs. These examples were selected because they represent common real-world challenges in traffic sign segmentation, including scale variation, cluttered backgrounds, and multiple adjacent traffic signs. The second column shows the predicted probability map, which is the raw continuous output of the network after sigmoid activation. Each pixel in this map has a value in the range [0, 1], where values close to 1 indicate high confidence that the pixel belongs to a traffic-sign region, and values close to 0 indicate background. Therefore, this column should not be interpreted as a final binary segmentation mask. Instead, it visualizes the confidence distribution learned by the model before thresholding. The third column shows the thresholded binary prediction, which is obtained by applying a fixed threshold of τ = 0.5 to the predicted probability map. This binary prediction is the final segmentation output used for quantitative evaluation, including Dice coefficient and IoU. As shown in Figure 4, the proposed model produces compact and localized traffic-sign masks for single signs, multiple nearby signs, and stacked signs, while suppressing most irrelevant background regions.

## 5. Conclusions and Future Directions

This paper presents R2KAN-U-Net, a Residual–Recurrent Kolmogorov–Arnold Network U-Net for robust traffic sign segmentation. The proposed architecture integrates residual convolutional learning, multi-scale KAN fusion, and recurrent KAN refinement to improve nonlinear feature representation and pixel-level foreground segmentation under challenging road-scene conditions. Experiments on the custom traffic-sign dataset and public benchmark settings demonstrate competitive segmentation accuracy, strong low-light robustness, and favorable computational efficiency. Ablation studies further confirm that the proposed components contribute complementary benefits to segmentation performance.

Future work will focus on lightweight deployment through pruning, quantization, and inference optimization, as well as broader validation under adverse weather and nighttime conditions. We also plan to investigate multimodal extensions using RGB, infrared, and LiDAR information and to release a representative dataset subset with annotation guidelines to support reproducibility.

## Figures and Tables

**Table 1:** Training configuration.

Optimizer	Adam with learning rate 1 × 10^−4^
Batch size	16
Epochs	400 with early stopping
Hardware	NVIDIA A100 GPU
Loss	Binary cross-entropy with Dice coefficient

**Table 2:** Comparison on our custom dataset.

Model	Dice Coefficient	IoU Score	Pixel Accuracy (%)
U-Net	0.82 ± 0.013	0.75 ± 0.015	94.5 ± 0.5
R2U-Net	0.84 ± 0.011	0.76 ± 0.013	96.4 ± 0.4
DeepLabV3+	0.84 ± 0.010	0.81 ± 0.012	96.2 ± 0.4
PSPNet	0.82 ± 0.012	0.80 ± 0.014	97.2 ± 0.5
SegFormer	0.89 ± 0.008	0.86 ± 0.010	97.9 ± 0.3
Mask2Former	0.90 ± 0.007	0.87 ± 0.009	97.1 ± 0.3
ConvNeXt V2 + UPerNet	0.88 ± 0.009	0.85 ± 0.011	97.5 ± 0.4
OneFormer	0.91 ± 0.006	0.88 ± 0.008	97.8 ± 0.3
SegMAN-S	0.90 ± 0.007	0.87 ± 0.009	97.6 ± 0.3
**R2KAN-U-Net (Ours)**	**0.92 ± 0.005**	**0.89 ± 0.007**	**98.4 ± 0.2**

**Table 3:** Quantitative robustness comparison under low-light conditions on the custom traffic-sign dataset.

Model	Overall IoU	Overall Dice	Low-Light IoU	Low-Light Dice
U-Net	0.75	0.82	0.61	0.73
DeepLabV3+	0.81	0.84	0.65	0.76
SegFormer	0.86	0.89	0.80	0.82
Mask2Former	0.87	0.90	0.73	0.83
ConvNeXt V2 + UPerNet	0.85	0.88	0.71	0.80
OneFormer	0.88	0.91	0.76	0.85
SegMAN-S	0.87	0.90	0.74	0.83
**R2KAN-U-Net (Ours)**	**0.89**	**0.92**	**0.79**	**0.88**

**Table 4:** Computational efficiency comparison with classical and contemporary segmentation models.

Model	Parameters (M)	FLOPs (G)	Inference Time (ms)
U-Net	31	57.4	12.5
DeepLabV3+	54	168.2	18.2
SegFormer	27	63.1	16.5
Mask2Former	47	229.6	25.0
ConvNeXt V2 + UPerNet	60	101.3	19.4
OneFormer	109	269.1	37.8
SegMAN-S	28	49.2	15.3
**R2KAN-U-Net (Ours)**	**24**	**44.8**	**13.0**

**Table 5:** Comparison study on ADE20K dataset.

Model	Dice Coefficient	IoU Score	Pixel Accuracy (%)
U-Net	0.741	0.621	93.7
R2U-Net	0.758	0.642	94.1
DeepLabV3+	0.782	0.671	94.8
PSPNet	0.773	0.662	94.5
Mask2Former	0.826	0.726	95.7
**R2KAN-U-Net (Ours)**	**0.842**	**0.744**	**96.1**

**Table 6:** Comparison on Cityscapes dataset.

Model	Dice Coefficient	IoU Score	Pixel Accuracy (%)
U-Net	0.861	0.732	97.2
R2U-Net	0.878	0.756	97.6
DeepLabV3+	0.891	0.774	97.9
**R2KAN-U-Net (Ours)**	**0.921**	**0.821**	**98.5**

**Table 7:** Ablation.

Model Configuration	Dice Coefficient	IoU Score	Pixel Accuracy (%)
**Full R2KAN-U-Net (Ours)**	**0.924**	**0.89**	**98.4**
No Recurrent Blocks	0.884	0.769	98.1
No Multi-Scale Fusion	0.892	0.777	98.2
No Residual Connections	0.876	0.753	98.2
No Recurrent Blocks + No Multi-Scale Fusion	0.861	0.741	97.8
No Residual Connections + No Recurrent Blocks	0.852	0.732	97.7

**Table 8:** Replacement ablation of the bottleneck/refinement module on the custom traffic-sign dataset.

Variant	Params (M)	Flops (G)	Time (ms)	Dice	IoU	Pixel Acc (%)
Residual U-Net baseline	13.5	28.4	7.8	0.871	0.748	97.9
Residual U-Net + MLP bottleneck	15.2	30.6	8.3	0.879	0.758	98.0
Residual U-Net + SE attention	13.8	29.1	8.1	0.885	0.764	98.1
Residual U-Net + CBAM	14.6	30.2	8.6	0.890	0.770	98.1
Residual U-Net + ConvNeXt-style block	16.3	33.5	9.4	0.895	0.776	98.2
Residual U-Net + SSM/Mamba block	17.8	36.2	10.1	0.901	0.783	98.2
Residual U-Net + KAN bottleneck	20.1	39.7	11.4	0.883	0.754	97.2
R2KAN-U-Net w/o recurrent refinement	21.9	42.3	12.1	0.884	0.769	98.1
**Full R2KAN-U-Net (Ours)**	**24.0**	**44.8**	**13.0**	**0.924**	**0.890**	**98.4**

## Data Availability

The data supporting the findings of this study are available from the corresponding author upon reasonable request.
